# Combining brain-computer interfaces with deep reinforcement learning for robot training: a feasibility study in a simulation environment

**DOI:** 10.3389/fnrgo.2023.1274730

**Published:** 2023-11-23

**Authors:** Mathias Vukelić, Michael Bui, Anna Vorreuther, Katharina Lingelbach

**Affiliations:** ^1^Applied Neurocognitive Systems, Fraunhofer Institute for Industrial Engineering (IAO), Stuttgart, Germany; ^2^Applied Neurocognitive Systems, Institute of Human Factors and Technology Management (IAT), University of Stuttgart, Stuttgart, Germany

**Keywords:** brain-computer interface, electroencephalography, event-related potentials (ERP), machine learning, deep reinforcement learning, robotics, error monitoring

## Abstract

Deep reinforcement learning (RL) is used as a strategy to teach robot agents how to autonomously learn complex tasks. While sparsity is a natural way to define a reward in realistic robot scenarios, it provides poor learning signals for the agent, thus making the design of good reward functions challenging. To overcome this challenge learning from human feedback through an implicit brain-computer interface (BCI) is used. We combined a BCI with deep RL for robot training in a 3-D physical realistic simulation environment. In a first study, we compared the feasibility of different electroencephalography (EEG) systems (wet- vs. dry-based electrodes) and its application for automatic classification of perceived errors during a robot task with different machine learning models. In a second study, we compared the performance of the BCI-based deep RL training to feedback explicitly given by participants. Our findings from the first study indicate the use of a high-quality dry-based EEG-system can provide a robust and fast method for automatically assessing robot behavior using a sophisticated convolutional neural network machine learning model. The results of our second study prove that the implicit BCI-based deep RL version in combination with the dry EEG-system can significantly accelerate the learning process in a realistic 3-D robot simulation environment. Performance of the BCI-based trained deep RL model was even comparable to that achieved by the approach with explicit human feedback. Our findings emphasize the usage of BCI-based deep RL methods as a valid alternative in those human-robot applications where no access to cognitive demanding explicit human feedback is available.

## 1 Introduction

In recent years, the technical capabilities and widespread use of autonomous and adaptive robots have increased enormously, expanding the application domain from traditional industrial contexts in areas such as medicine, domestic environments, health care, and entertainment (Yang et al., 2018; Hentout et al., 2019; Henschel et al., 2020). This has led to rising interest in research on how we can improve human-robot collaboration in general and how human feedback (HF) can be given during the learning of complex tasks. Sophisticated algorithms such as deep reinforcement learning (RL) can be used to teach robotic agents how to autonomously learn new complex skills (Mnih et al., 2015). Learning is based on interaction with the environment in a process of trial and error. An important element of RL is the policy that defines the learning of the agent's behavior at a given time and corresponds to the mapping of observed states to actions (Sutton and Barto, 2018). For the agent to learn an optimal policy, it is essential that feedback can be defined in the form of a good reward function (criticism and reward). Providing feedback during the initial stages of learning is crucial to facilitate the exploration of promising behaviors early on. The reward function delineates the goal within a RL problem, elucidating what constitutes favorable or unfavorable behavior for the agent (Sutton and Barto, 2018).

Yet, the derivation or design of a suitable reward function remains a major challenge (Xavier Fidêncio et al., 2022), especially in real-world scenarios. In such scenarios, the agent usually faces the problem of sparse extrinsic rewards, so-called *sparse reward environments*. These environments are characterized by a small number of states that provide a positive feedback signal for the agent. Furthermore, sparsity is a natural way to define a reward in a real-world scenario (Kober et al., 2013; Riedmiller et al., 2018; Singh et al., 2019). The agent exclusively receives a positive reward upon completing the task or achieving the final goal, without receiving any rewards for intermediary stages. Consequently, sparsity provides few learning signals for the agent. In addition, the probability of the agent accidentally achieving the goal or completing the task is extremely low. This makes state-of-the art deep RL from sparse rewards—without additional mechanisms to learn the optimal balance of exploitation and exploration—very time-consuming or sometimes even impossible. Furthermore, possible feedback given by the human during learning is often not considered in the reward settings or function.

The simplest solution to design a reward function is *reward shaping* (Wiewiora, 2003; Grzes and Kudenko, 2009). Reward shaping, however, firstly requires a huge amount of domain knowledge, e.g., by a human expert, about the task to be solved. In a second step, the domain knowledge must be converted into explicit machine-understandable instructions. Learning such a reward function is, therefore, a very tedious and iterative process, which requires explicit expert knowledge. Alternatively, demonstrations can be used to initiate, guide, and reinforce certain behavior during learning—so-called *learning from demonstrations* (Blau et al., 2021; Pertsch et al., 2021). While this can be a very simple and effective method, it requires that the task is first explicitly displayed by the human, which is not always possible, e.g., in human-robot collaboration.

A very intuitive and attractive alternative to overcome weaknesses of reward shaping and learning from demonstrations is the use of interactive RL (Kim et al., 2017) or more generally speaking *learning from human feedback* (Suay and Chernova, 2011; Grizou et al., 2013; Christiano et al., 2017; Warnell et al., 2017). In a supervised manner, the human evaluates the actions of the agent as it learns behavior in certain states. During the agent's learning the human can classify single states as good or bad, thus fostering the agent to reinforce those actions that are classified as good.

In recent years many techniques have been proposed to estimate given HF using either speech or gesture recognition from eye, body or head tracking (Yip et al., 2016; Takahashi et al., 2017; Mittal et al., 2020). However, these methods alone are not specific enough and they depend on explicitly expressed human cognitive behavior. More specifically, speech or gestures can be ambiguous, or they may increase the mental load of the users. Both require explicit instructions and verbal communication which may further lead to distractions in the execution of the user's task of interest. Steady progress in the development of sensor technologies including miniaturization and mobile use, coupled with advanced signal processing and machine learning, allows us to derive many facets of subtle mental user states, like attention, cognitive load, or error perception from brain signals (Blankertz et al., 2016; Cinel et al., 2019; Vukelić, 2021; Niso et al., 2022; Roy et al., 2022). While research in brain-computer interfaces (BCIs) has focused mainly on medical and clinical applications (Carlson and Millan, 2013; Ramos-Murguialday et al., 2013; Brauchle et al., 2015; Leeb et al., 2015; Kern et al., 2023), more and more attention is now directed toward monitoring diverse activities in real-world related scenarios, thus laying the basis for non-medical applications of BCIs (Blankertz et al., 2016; Cinel et al., 2019; Vukelić, 2021).

Passive or implicit BCIs (Zander and Kothe, 2011) are particularly important for teaching robots complex skills. They enable the use of immediate and implicit human reactions or impressions as feedback (Perrin et al., 2010; Zander et al., 2016; Edelman et al., 2019; Iwane et al., 2019). Making a mistake or observing a mistake being made—even by a robotic agent—elicits an error-related potential (ErrP) which can be measured using electroencephalography (EEG). ErrPs are predominantly observed over frontocentral regions in the EEG and characterized by three main components in the averaged time courses when comparing errors to correct actions. The components are a negative deflection occurring around 200 ms called N200, a positive deflection at around 300 ms called P300, and another negative deflection at around 400 ms referred to as N400 (Chavarriaga et al., 2014; Iturrate et al., 2015; Spüler and Niethammer, 2015; Ehrlich and Cheng, 2019).

Since human error perception is closely coupled with learning mechanisms, the use of this error recognition is particularly suited for reinforcement learning (Iturrate et al., 2015; Kim et al., 2017). Even if the reaction to errors differ between certain tasks (motor or more abstracts), it is still universally recognizable using machine learning (Chavarriaga et al., 2014; Spüler and Niethammer, 2015; Wirth et al., 2020). The human can observe and implicitly evaluate the value of an action performed in the respective state. The feedback given is thus very direct and fast, without extra effort on the part of the human. Previous approaches using decoded ErrPs as a feedback signal for reinforcement learning were either real-time—i.e., the human had to provide feedback during the whole learning processes—or had rather simple, mainly discrete RL state spaces as test environments—e.g., small 1-D cursor movements or 2-D discretized state spaces of robot reaching tasks— (Iturrate et al., 2013, 2015; Zander et al., 2016; Kim et al., 2017; Luo et al., 2018; Schiatti et al., 2018; Ehrlich and Cheng, 2019).

Recently, Akinola et al. (2020), employed the use of ErrP-decoded signals indirectly in an RL environment and combined it with a more sophisticated on-policy deep RL algorithm called proximal policy optimization (Schulman et al., 2017). The proposed algorithm (BCI + deep RL) consists of three stages: (1) Calibration of an EEG-based BCI for the automatic recognition of perceived errors, (2) estimation of a HF policy (approximation of a fully connected neural network in real-time) based on implicit feedback through the BCI, and (3) learning a final RL policy strategy from sparse rewards in which the HF policy guides the RL policy exploration at the beginning. Interestingly, the approach accelerated the early learning during a simple navigation task in a discretized action space problem and achieved a stable performance once the HF was no longer available. Minimizing human involvement during learning is an attractive approach for real-world human-robot collaboration tasks, which warrants further research.

In the context of our long-term perspective, our primary aim is to enhance the practical utility of BCIs by employing dry-based EEG systems. Building upon Akinola et al. (2020), this research seeks to systematically expand upon their work in two distinct ways: (1) Demonstrating the feasibility of decoding ErrP-based implicit user reactions in a physically realistic 3-D continuous robot simulation environment comparing a mobile dry-based and gel-based EEG system with different channel number configurations. The evaluation of dry-based EEG for ErrP classification, compared to gel-based systems, provides a practical solution to streamline setup procedures. Consequently, we address a notable gap in the literature as comprehensive studies benchmarking the performance of dry-based EEG systems specifically for ErrP classification are limited. (2) Comparing *implicitly* (rating of robot behavior using a BCI) and *explicitly* (rating of robot behavior was recorded directly via keyboard input) trained HF policies in this realistic simulation environment.

## 2 Materials and methods

### 2.1 Participants

Twenty-two volunteers (*M*_*age*_ = 29.35, *SD* = 4.59 years, 9 female and 13 male participants) were recruited and divided into two studies. Participants gave their written informed consent before participation and received monetary compensation. The study protocol was approved by the Local Ethics Committee of the Medical Faculty of the University of Tuebingen, Germany (ID: 827/2020BO1).

### 2.2 General study design

#### 2.2.1 Study one

In the first experiment (*N* = 16 participants), we pursued two objectives: (1) To investigate the classification performance of two machine learning models, a Riemannian geometry-based classifier and a convolutional neural network (CNN) classifier. Both models have demonstrated sufficient performance in motor imagery (Schirrmeister et al., 2017; Lawhern et al., 2018; Al-Saegh et al., 2021) or attentional processes via P300 (Yger et al., 2017; Delgado et al., 2020; Li et al., 2020). The models were mostly studied for active or reactive BCI decoding performance (Lawhern et al., 2018; Appriou et al., 2020) but were not systematically compared for decoding ErrP in a realistic robot simulation environment and with a dry-based EEG system. We, therefore, were also interested in (2) the influence of channel number and EEG research system on the classification performance (gel-based vs. dry-based). As a benchmark assessing classification performance a conventional approach was employed in the form of statistical feature extraction in the time domain and two multivariate conventional linear classifiers: Linear discriminant analysis (LDA) and support vector classification (SVC). To investigate the influence of the EEG research system on the BCI classification performance, a high standard mobile gel-based (64-channel actiCAP slim system and LiveAmp 64 wearable 24-bit amplifier from BrainProducts GmbH) was compared with a high standard mobile dry-based EEG system (CGX Quick-20r from Cognionics Inc.). We collected data from nine participants with the gel-based EEG system and from seven participants using the dry-based EEG system.

#### 2.2.2 Study two

In the second feasibility study (*N* = 6 participants), the difference between an implicitly trained version of an HF policy function was compared to an explicitly trained one. Thereby, we extended the approach of Akinola et al. (2020) who contrasted a sparse reward function (RL sparse) and a richer reward function (RL rich). The sparse reward function only provided positive feedback for reaching the target, while the rich reward function extended the sparse formulation by including additional informative reward with the Euclidean distance from the goal and current position. We implemented two versions of an HF policy for the BCI + deep RL algorithm: (1) A policy allowing implicit BCI-based given feedback and (2) a policy allowing explicitly given feedback (keyboard button press, “*y*” for correct and “*n*” for incorrect behavior). Three of the six participants trained the HF policy function with the implicit BCI version and the remaining three with the explicit one.

#### 2.2.3 Robot simulation environment, trial, and task procedure

**Figure 1:**
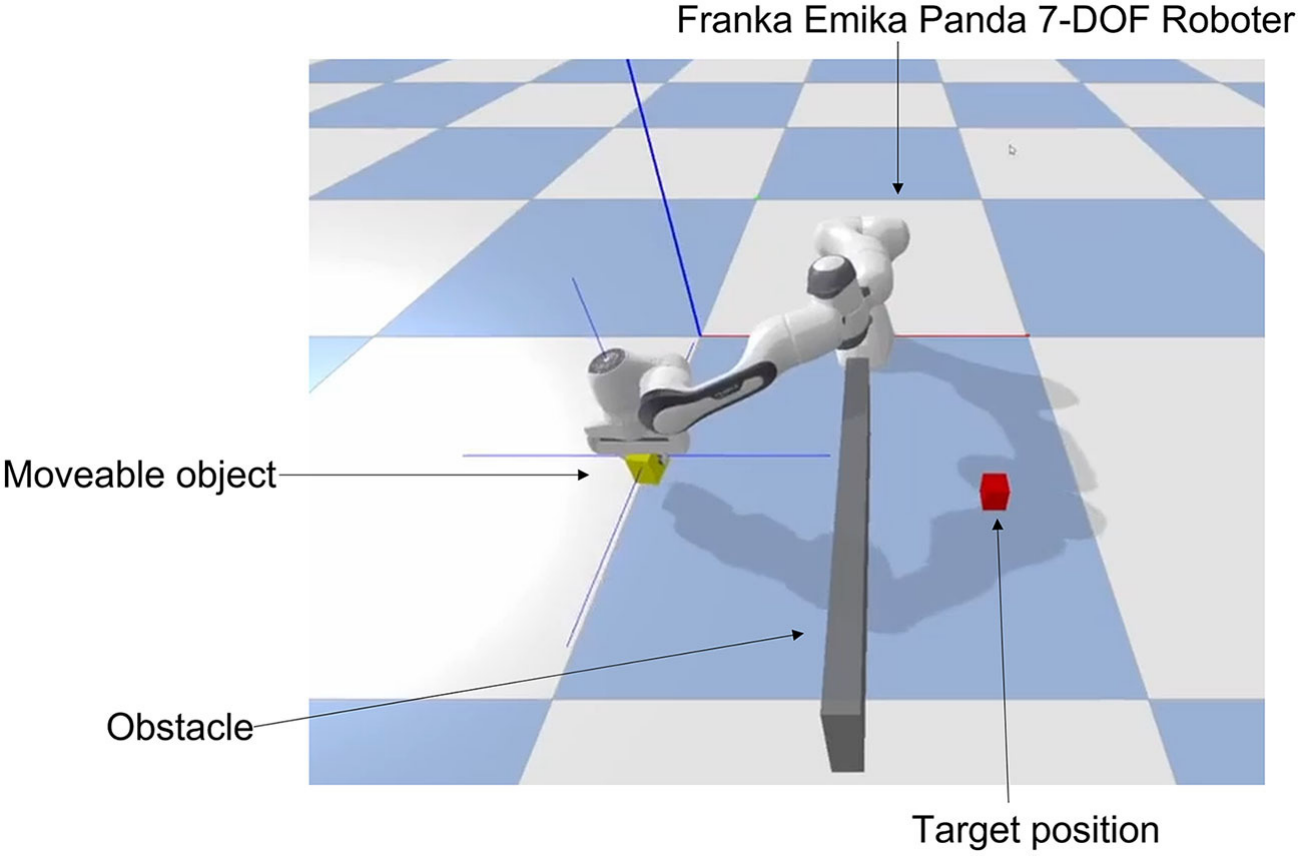
The simulation environment in Bullet and Pybullet which can be used in OpenAI Gym. In this gripping and navigation task the robot agent (Franka Emika 7-DOF robot) moves its end effector to place the yellow object (moveable object) at the target (red object). The yellow object starts at a random position after each run, while the robot arm starts at the current position of the yellow object. The task is to navigate the yellow object to the red target object while avoiding self-collision and collision with the wall (obstacle). The position of the red target object changes randomly after each run. The challenge is to avoid the obstacle wall and collisions with the robot arm to reach the goal on the shortest path possible. As quickly and efficiently as possible.

In our work, we utilized a 3-D physically realistic open-source simulation environment implemented with Bullet Physics SDK.^1^ Bullet Physics SDK provides a fast and easy-to-use library in Python—PyBullet—for robotics, virtual reality, and reinforcement learning as well as suitable simulation environments, e.g., KUKA or Franka robotic agents. Thus, realistic simulations of forward and inverse dynamics and kinematics as well as collision detection can be realized. Furthermore, the API offers the possibility to implement common machine learning environments like OpenAI Gym,^2^ TensorFlow^3^ and Pytorch^4^ and to explore sophisticated deep RL algorithms for learning complex robot skills. The task to be learned by participants was presented in a virtual environment (see Figure 1). We used a Franka Emika Panda 7-DOF robot agent as a continuous action/state space environment. To facilitate the participant's assessment with an EEG-based BCI, we have modeled the RL problem with a discrete action space and defined six actions: Moving left, right, forward, backwards, down, and up. The state-space consists of a 3D vector in cartesian coordinates, where the continuous values are clipped into a discrete grid area with dimensions 21 × 21 × 11, and five laser sensor observations. The state space output values are normalized in a range from 0.0 to 1.0. Detailed definitions of the environment and its use in OpenAI Gym are provided in Supplementary Figures S1–S3.

**Figure 2:**
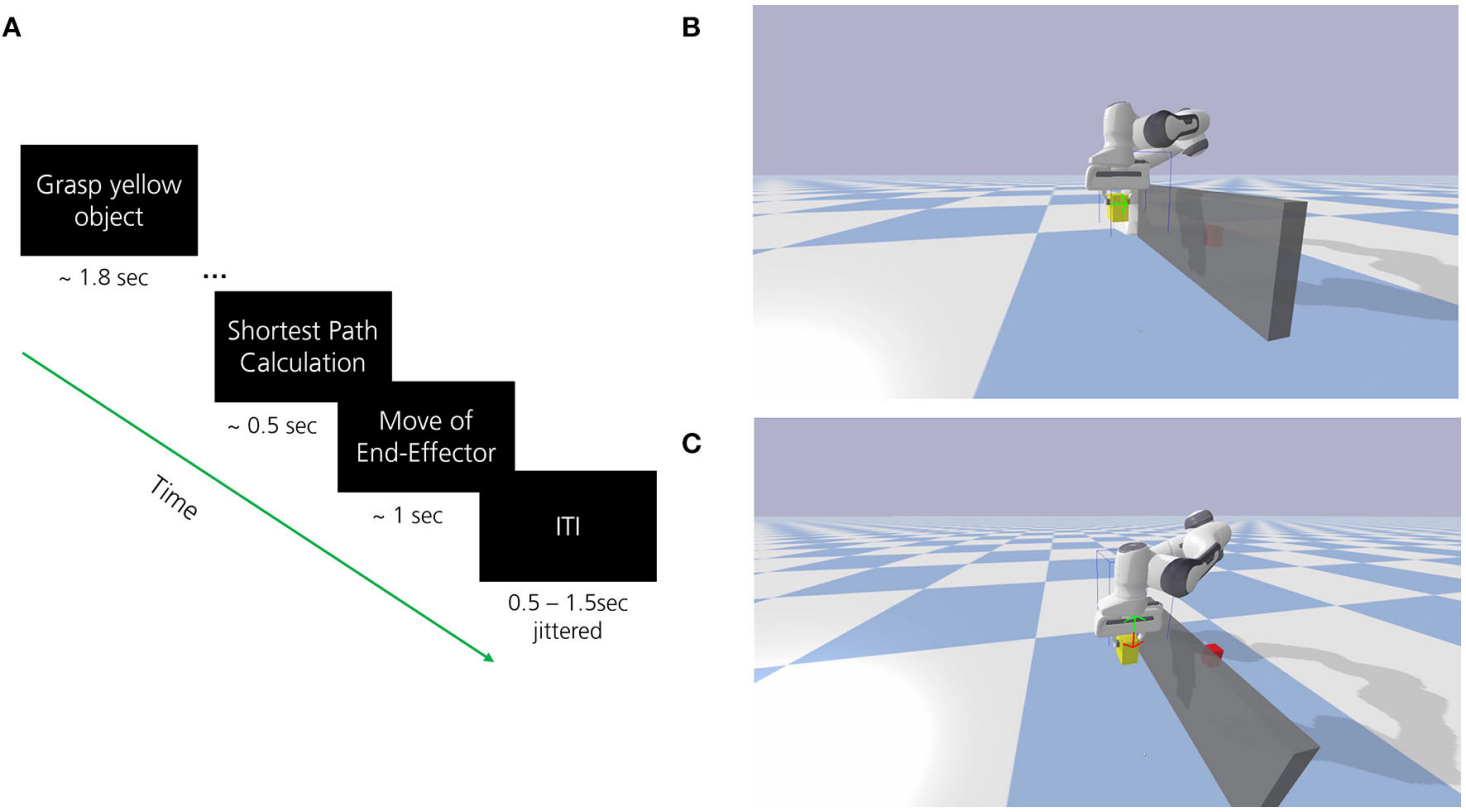
**(A)** The event-related trial procedure to decode observed errors in robot behavior with EEG. The shortest path from start (yellow moveable object) to goal (red target object) has been defined as the optimal path for robot behavior. The participant observed the robot behavior and mentally evaluated whether the robot performed the intended behavior (indicated by the direction of movement of the green arrow). **(B)** Depiction of situation where the end effector navigates to the right (shortest path) as supposed to (correct or optimal robot behavior, green arrow). **(C)** Depiction of situation where the end effector navigates downwards (incorrect or suboptimal robot behavior, red arrow) as opposed to upwards (green arrow). Please note that only the green arrow was shown prior to the end effector movement during the calculation of the shortest path to give information on the intended robot behavior, the red arrow is only shown for illustration purpose.

In all experiments, participants were instructed to observe and mentally evaluate the performance of the navigation steps performed by the robot. The robot attempted to move a yellow block toward a target (red block) using the optimal path while avoiding self-collision or collision with obstacles (see Figure 1). The optimal path was determined by calculating the shortest path from each given state to the goal position using the A^*^ search algorithm. This path, represented by a green arrow, indicated the correct and intended robot behavior. Thus, a correct action required the direction congruent to the one signaled by the green arrow (see Figure 2B). An incorrect performance was defined as an action with a direction incongruent to the green arrow (see Figure 2C). The goal position remained fixed during each run and the start position of the yellow block was randomly set once the agent the run was finished. Each episode started with the robot grasping the yellow block which was randomly placed within a 6 × 11 grid area. The event-related trial procedure is displayed in Figure 2A. The developed environment included a Python-based connection to the Lab Streaming Layer (LSL) for the acquisition and synchronization of the simultaneously recorded EEG data and marker labels for the trial events. To ensure signal quality during data collection, participants were further asked to limit eye movements, blinking, and possible teeth grinding as much as possible to the indicated breaks.

## 3 Study one

### 3.1 Data collection

In study one, we recorded EEG of 500 single robot movements per participant with a probability of 20% for erroneous actions resulting in a total of 100 erroneous robot actions (Iturrate et al., 2015). For the gel-based system, we recorded scalp EEG potentials from 64 positions (placed according to the extended international 10-05 system) using Ag/AgCl electrodes. The left mastoid was used as a common reference and EEG was grounded to Cz. All impedances were kept below 20 kΩ at the onset of each session. EEG data were digitized at 250 Hz, high-pass filtered with a time constant of 10 s and stored for offline data analysis using LSL. For the dry-based EEG system, we recorded scalp EEG potentials from 20 positions (placed according to the international 10-20 system) using DryPad and FlexSensors of the CGX Quick-20r system. EEG data were digitized at 500 Hz, high-pass filtered with a time constant of 10 s and stored for offline data analysis using LSL.

In the first analysis step before the classification analysis, we combined the EEG data across all participants and epochs to visually explore the correlates during the perception of optimal (true) and suboptimal (error) robot behavior for each EEG system. We calculated the grand average per condition over midline frontal (Fz) and central electrodes (C3, Cz, and C4) (see Iturrate et al., 2015; Spüler and Niethammer, 2015). Furthermore, to allow comparisons with previous research results, we included the grand average per condition over the fronto-central electrodes (FC1 and FC2) only for the gel-based EEG. It is important to note that these electrodes are not present in the montage of the dry-based EEG. However, they are commonly reported for ErrPs (Spüler and Niethammer, 2015; Kim et al., 2017; Wirth et al., 2020).

We further compared the signal-to-noise ratio (SNR) over the frontal and central electrodes of the two EEG systems. The SNR was calculated separately for the N200 (with a time interval ranging from 100 to 300 ms after action onset) and a delayed P300 (with a time interval ranging from 300 to 600 ms after action onset). We computed the SNR on a subject level using the contrast “error vs. correct actions” (averaged signals across epochs). For the SNR calculation, the amplitude within the ErrP time interval was divided by the standard deviation of the ErrP time interval, which served as a representation of the noise amplitude (Hu et al., 2010).

To compare the SNR of the two EEG systems, we used bootstrapping with 5,000 iterations to calculate a mean and its 95% confidence interval (CI) for each approach, ErrP, electrode position and EEG system. Bootstrapped means and their CIs offer the possibility to make statistical statements about possible differences (Cumming and Finch, 2005). No overlap of the bootstrapped means' CIs indicate a strong statistical significance (*p* < 0.01) and a partial overlap without inclusion of the mean indicates a moderate statistical significance of *p* < 0.05 (Cumming and Finch, 2005).

### 3.2 Machine learning for decoding error perception

Altogether, we compared three EEG conditions—gel-based EEG with (1) 64 channels, (2) 16 channels (channels were selected based on Iturrate et al., 2015), and (3) dry-based EEG with 20 channels—and four machine learning approaches. The four machine learning approaches were: Feature extraction in combination with two conventional multivariate linear classifiers (LDA and SVC), classification based on Riemannian geometry (Riemannian-based classifier) (Appriou et al., 2020) and the CNN-based classifier EEGNet (Lawhern et al., 2018). In all approaches, supervised learning was performed per participant to classify optimal (true) and suboptimal (error) robot behavior. For the python implementation of the classifiers, we used the following libraries: scipy, numpy, mne including mne-features, scikit-learn, pyRiemann, and TensorFlow Keras.

#### 3.2.1 EEG pre-processing

Before classification, we pre-processed the EEG data according to the proposed pipeline of Iturrate et al. (2015). Pre-processing was the same for both the gel- and dry-based EEG systems. In the first step, all trials of the optimal (true) and suboptimal (error) robot behavior were grouped. Next, the EEG signals were detrended, zero-padded, and digitally filtered using a power-line notch filter at 50 Hz (IIR filter with filter order of 4) followed by a band-pass filter at [1, 10] Hz (IIR Butterworth filter with filter order of 4). Afterwards, we spatially filtered the EEG signals using a common average reference and finally downsampled the data to 250 Hz (only for the dry-based EEG) to have the same sampling rate for both systems. Next, we split the continuous EEG signals into stimulus-locked (i.e., the onset of the end-effector movement) segments of 1.2 s, consisting of a 200 ms baseline (before onset, −0.2 to 0 s) and a 1 s after the end-effector movement of the robot. For each participant, all stimulus-locked segments were aligned by subtracting the average value of the baseline from the remaining time window. For all machine learning models, we focused on the following time window of interest: 200–800 ms after the robot end-effector movement.

#### 3.2.2 Feature extraction and conventional machine learning models

In the next step, we extracted time domain features from all possible EEG channels of the gel- and dry-based EEG data. For each class sample (true and error) and participant, we extracted the following features from the time window of interest: Mean amplitude, skewness, kurtosis, standard deviation, and peak-to-peak amplitude using the mne-features API *FeatureExtractor*. Next, we explored the LDA and SVC machine learning model as implemented in the scikit-learn machine learning package (version 0.22.2). First, we re-scaled the features using the *StandardScaler* implemented in scikit-learn, to ensure that for each feature the mean is zero and to scale to unit variance, thereby bringing all features to the same magnitude. Next, we only kept the most meaningful features in the data by applying a principal component analysis (PCA) and selecting those components explaining 95% of the variance in their sum when ranked decreasingly based on their contribution.

We optimized the hyperparameters for each classifier individually. For the LDA, the solver function (singular value decomposition, least squares solution, or eigenvalue decomposition) was adjusted and for the SVC, the strength of the regularization and kernel coefficient of the radial basis function was applied. We performed the hyperparameter optimization with a 5-fold cross-validated grid search (*GridSearchCV*, inner loop, 5 splits). The quality of each model was assessed using a repeated stratified *k*-fold cross-validation (*RepeatedStratifiedKFold*, outer loop, 5 splits, and 10 repeats) and the area under the receiver operating characteristic curve (ROC-AUC) as metric.

#### 3.2.3 Riemannian geometry-based model

The Riemannian-based method does not require feature extraction but works directly with the time series of the pre-processed and epoched EEG signals. As above, we focused on the time window of interest, for training the classifier and evaluation of its performance. As described in detail by Appriou et al. (2020), Riemannian approaches represent epoched EEG signals as symmetric positive definite (SPD) covariance matrices and manipulate them with a suitable Riemannian geometry (Congedo et al., 2017). Generally, Riemannian geometry deals with uniformly curved spaces that behave locally like Euclidean spaces. To apply the Riemannian approach to our data we used the *pyRiemann* python library. In the presented Riemannian manifold, covariance matrices of event-related potentials were estimated and spatially filtered based on the xDAWN algorithm (Rivet et al., 2009). Subsequently, the covariance matrices were projected into the tangent space for a detailed description see (Barachant et al., 2012). The tangent space projection is useful to convert covariance matrices into Euclidean vectors while preserving the inner structure of the manifold. After this projection, the classification was applied (Appriou et al., 2020). For classification, we coupled the Riemannian-based approach with an LDA classifier (using the default settings with singular value decomposition as solver) without hyperparameter optimization. To validate the model quality, a repeated stratified *k*-fold was again employed (*RepeatedStratifiedKFold*, outer loop, 5 splits, and 10 repeats) with the ROC-AUC as metric.

#### 3.2.4 Deep learning model—convolutional neural network

Similar to the Riemannian-based classifier, no explicit feature extraction is needed in the deep learning approach. The model can directly be applied to the pre-processed and epoched EEG time series. For classification, we focused again on the time window interest. We utilized a modified version of EEGNet (Lawhern et al., 2018) as implemented in Keras (v.2.2.4). EEGNet employs depth-wise convolution and separable convolution layers (Chollet, 2016). The convolution operates along the temporal and spatial dimensions of the EEG signal. The EEGNet architecture consists of three blocks. In the first block, two convolutional steps are performed for optimizing bandpass filters (temporal convolution), followed by a depth-wise convolution to optimize frequency-specific spatial filters (Schirrmeister et al., 2017; Lawhern et al., 2018). The second block involves the use of separable convolution which reduces the number of parameters to fit in the network (Lawhern et al., 2018). The output of the second block is fed directly to a third classification block with a softmax activation function. The configuration parameters were implemented according to Lawhern et al. (2018): The number of channels was 64 or 16 for the gel-based and 20 for the dry-based EEG system, the number of classes was 2, the number of temporal filters was 8, the number of pointwise filters was 16, the number of spatial filters was 2, the kernel length was equal to the sampling rate divided by 2. To deal with model instability and potential overfitting we used dropout (rate of 0.5) as a regularization strategy in combination with exponential linear units (ELU) and batch normalization (Schirrmeister et al., 2017; Lawhern et al., 2018). Categorical cross-entropy was used as a loss function with the Adam optimizer (initial learning rate was 0.01 and mini-batch size was 16). To further improve model generalization and stability we used a plateau-based decay strategy. Once the learning stagnated, the learning rate was reduced by a factor of 10 when the validation loss stopped improving for five consecutive epochs. To validate the model quality, a repeated stratified *k*-fold from scikit-learn was used (*RepeatedStratifiedKFold*, outer loop, 5 splits, and 10 repeats) and the ROC-AUC as metric. For each *k*-fold, we trained the model with 300 training epochs.

#### 3.2.5 Statistical comparison of machine learning models

To assess the stability of the model's performance (generalization capabilities) and the uncertainty or variability associated with its prediction we estimated a distribution of the average performance (ROC-AUC) from the training and test data sets per classifier via bootstrapping (5,000 iterations). This was done over single folds and repetitions of the repeated stratified *k*-fold cross-validation. Calculating the mean and its 2.5th and 97.5th CI from this distribution also offers the possibility to make statistical statements about possible differences in performance (Cumming and Finch, 2005). The CIs were Bonferroni-corrected for multiple comparisons.

### 3.3 Results first study

**Figure 3:**
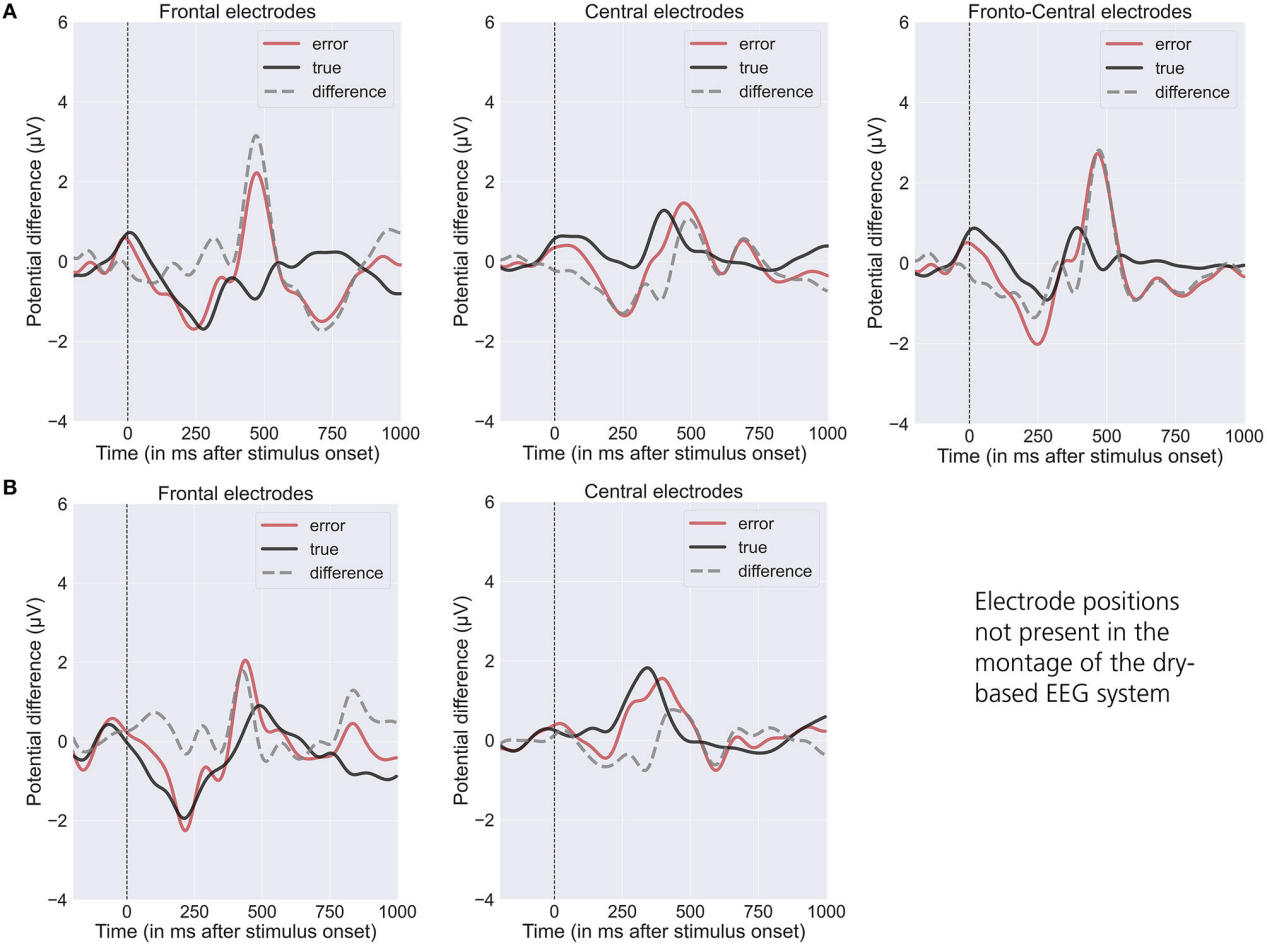
Grand averaged waveform (“error” = red line and “true” = black line) and differences (“error” minus “true” = gray line) of the event-related potentials elicited by the observation of the robots' end-effector movements (*t* = 0: Onset of end-effector movement) for the gel-based (**A**; *N* = 9) and dry-based EEG system (**B**; *N* = 7). Mean value over frontal (Fz; left), central (C3, Cz, and C4; middle), frontocentral (FC1 and FC2; right). EEG positions (note that frontocentral positions were only available for the gel-based EEG system).

The grand averages of event-related potentials associated with optimal and suboptimal actions of the robot exhibit a characteristic temporal pattern, displaying distinguishable differences in frontal (see Figures 3A, B, left), central (see Figures 3A, B, middle) and frontocentral (see Figure 3A, right) channels (Chavarriaga et al., 2014; Iturrate et al., 2015; Spüler and Niethammer, 2015; Kim et al., 2017; Ehrlich and Cheng, 2019). We observe an ErrP-related difference between the conditions ~200 ms after action onset followed by a late positive deflection at ~500 ms (see also Kim et al., 2017).

**Figure 4:**
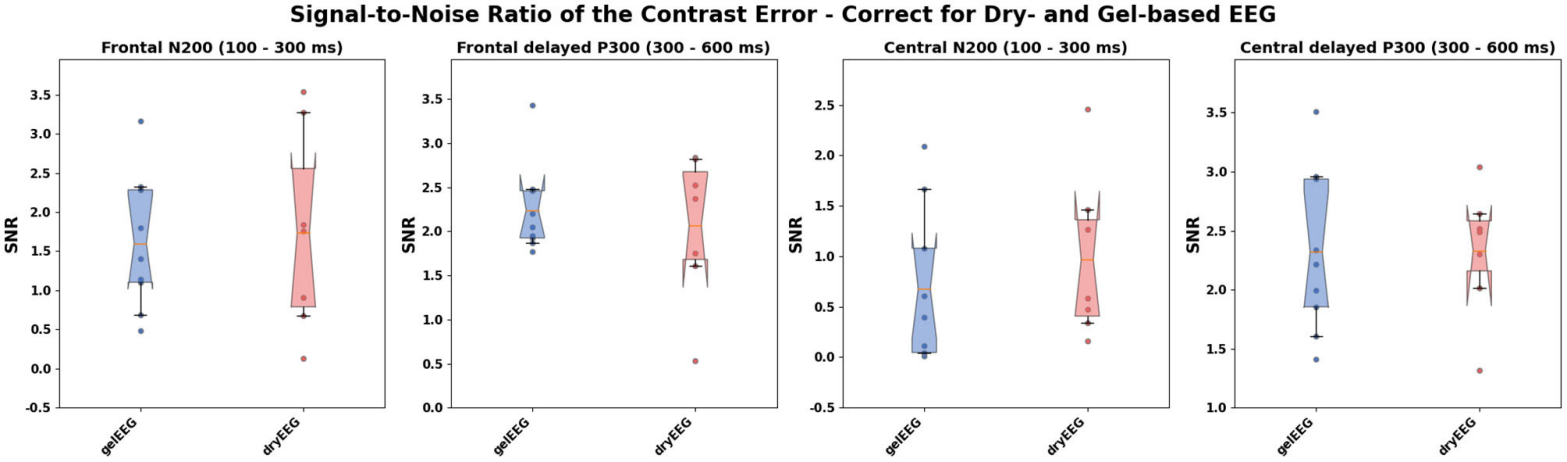
Distribution of the bootstrapped subject-wise signal-to-noise ratio (SNR) of the contrast error (suboptimal) vs. true (optimal) over the frontal (upper row) and central (lower row) electrodes of the gel-based (blue) and dry-based (red) EEG systems. The SNR was calculated separately for the N200 (with a time interval ranging from 100 to 300 ms after action onset; **left**) and a delayed P300 (with a time interval ranging from 300 to 600 ms after action onset, **right**) as suggested by Hu et al. (2010). Filled points per boxplot: SNR per subject. Whiskers of the boxplot indicate the 5th and 95th quartile of the distribution. The box comprises 50% of the distribution from the 25th to the 75th quartile. Notches in the boxes visualize the Bonferroni-corrected upper and lower boundary of the mean's 95% confidence interval (CI) used as statistical index. The solid orange line within the boxplot indicates the bootstrapped mean.

The SNR analysis revealed similar results for the dry-based and gel-based EEG systems in both ErrP time intervals (N200 and delayed P300) and electrode positions (frontal and central; see Table 1; Figure 4) with no statistically significant difference between the two EEG systems (Table 1).

**Table 1:** Statistical comparison of the signal-to-noise ratio between the dry- and gel-based EEG system at the different event-related potential time intervals and electrode positions.

	**Dry-based EEG**			**Gel-based EEG**		
**Electrode position and time interval**	**Lower CI**	**Bootstrapped means**	**Upper CI**	**Lower CI**	**Bootstrapped means**	**Upper CI**
Fz—N200	0.77	1.73	2.76	1.01	1.60	2.25
Fz—P300	1.95	2.24	2.65	1.34	2.06	2.63
C3, Cz, C4—N200	0.42	0.96	1.69	0.18	0.67	1.25
C3, Cz, C4—N200	1.87	2.33	2.72	1.85	2.32	2.82

**Figure 5:**
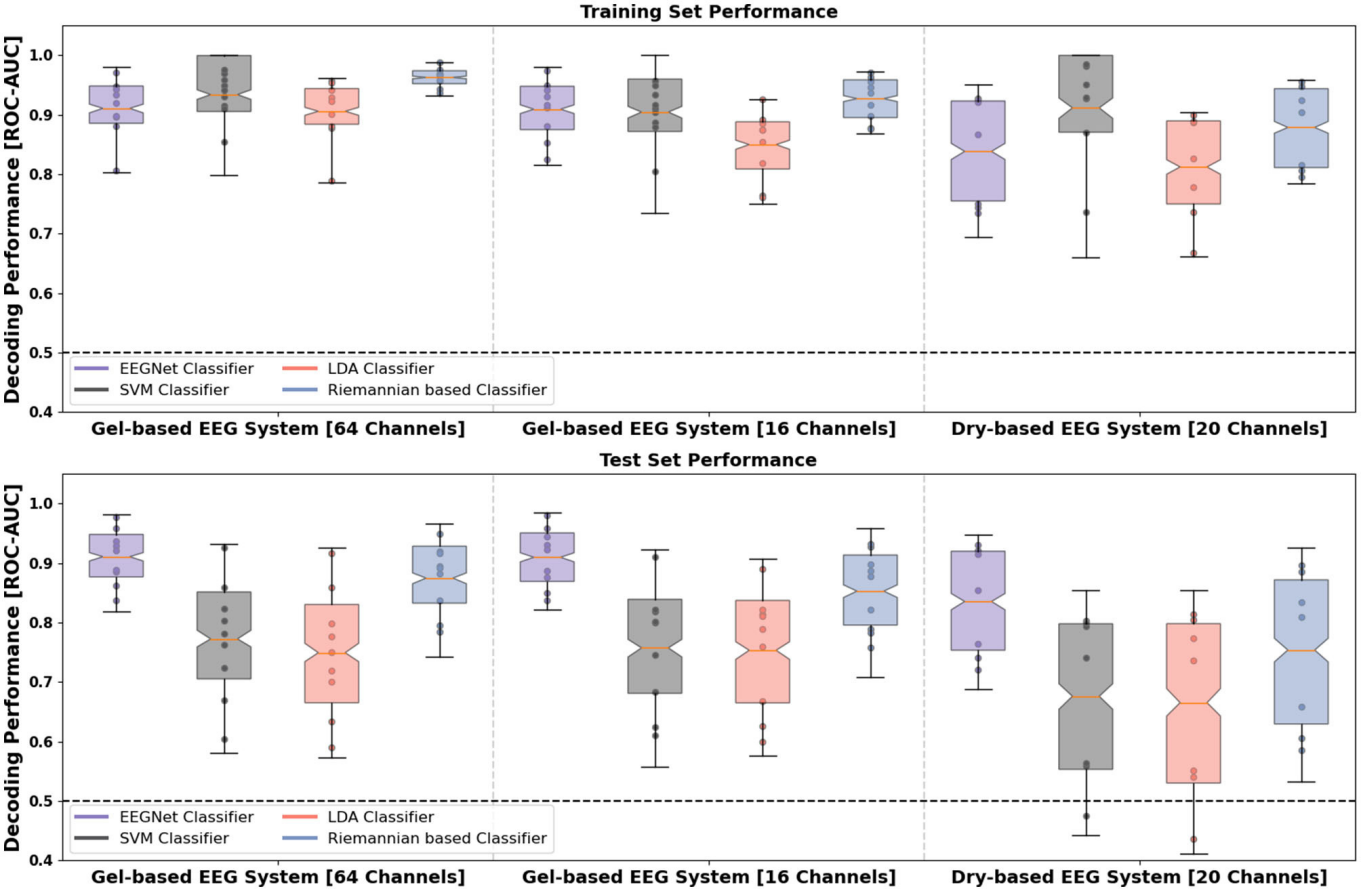
Distribution of the ROC-AUC decoding performance on the training **(upper row)** and test data sets **(lower row)** per subject (filled points per boxplot) and classification model. Whiskers of the box indicate the 5th and 95th quartile of the distribution. The box comprises 50% of the distribution from the 25th to the 75th quartile. Notches in the boxes visualize the Bonferroni-corrected upper and lower boundary of the mean's 95% confidence interval (CI) used as statistical index. The solid orange line within the boxplot indicates the bootstrapped mean. The dashed line shows the theoretical chance level (0.5 for a two-class classification problem). **Left:** Gel-based EEG with 64 channels; **Middle:** Gel-based EEG with 16 channels; **Right:** Dry-based EEG. Colors show the different machine learning models: Purple (EEGNet), red (linear discriminant analysis classifier), black (support vector classifier) and blue (Riemannian-based geometry coupled with LDA classifier). LDA, linear discriminant analysis; DL, deep learning.

Next, we assessed the feasibility of leveraging the distinct temporal waveform differences between the robot actions in various machine learning methods. We compared the classifications when using different channel number configurations in the gel-based EEG system (64 channels vs. 16 channels) as well as when using data obtained from the gel-based and dry-based EEG systems. Overall, above chance-level performance (the theoretical chance level at 0.5 for binary classification) was observed for all channel number configurations, EEG systems and four classifier models as estimated by the bootstrapped mean ROC-AUC accuracy as well as its 95% CI over single folds and repetitions of the repeated stratified *k*-fold cross-validation (see Figure 5).

Results of bootstrapped mean ROC-AUC on the test set demonstrate that the EEGNet model performed best with a performance of 0.911 [95% CI (0.904, 0.918)] for 64-channel, 0.910 [95% CI (0.902, 0.917)] for the 16-channel gel-based EEG, and 0.836 [95% CI (0.822, 0.850)] for the dry-based EEG system (see Table 2). The EEGNet model not only outperformed the two conventional multivariate linear classifiers (LDA and SVM) but also the Riemannian-based classifier for both EEG systems [dry-based: 0.754, 95% CI (0.734, 0.773)] and channel number configurations [64-channel: 0.875, 95% CI (0.866, 0.884) and 16-channel: 0.853, 95% CI (0.842, 0.864)]. The performance of the EEGNet did not significantly differ between the channel number configurations.

**Table 2:** Statistical comparison of machine learning models for the classification of optimal and suboptimal robot behavior from EEG data.

	**Training set**			**Test set**		
	**Lower CI**	**Bootstrapped means**	**Upper CI**	**Lower CI**	**Bootstrapped means**	**Upper CI**
**Gel-based EEG**−**64 channels**						
Linear discriminant analysis classifier	0.899	0.906	0.913	0.734	0.749	0.764
Support vector classifier	0.926	0.934	0.942	0.759	0.773	0.787
Riemannian based classifier	0.961	0.963	0.965	0.866	0.875	0.884
EEGNet classifier	0.904	0.911	0.918	0.904	0.911	0.918
**Gel-based EEG**−**16 channels**						
Linear discriminant analysis classifier	0.843	0.850	0.857	0.739	0.753	0.768
Support vector classifier	0.894	0.905	0.914	0.743	0.757	0.771
Riemannian based classifier	0.922	0.927	0.932	0.842	0.853	0.864
EEGNet classifier	0.903	0.909	0.916	0.902	0.910	0.917
**Dry-based EEG**−**20 channels**						
Linear discriminant analysis classifier	0.799	0.812	0.824	0.643	0.665	0.689
Support vector classifier	0.894	0.912	0.928	0.654	0.676	0.697
Riemannian based classifier	0.868	0.879	0.889	0.734	0.754	0.773
EEGNet classifier	0.824	0.838	0.852	0.822	0.836	0.850

The values show the mean ROC-AUC score from 50-folds and 5,000 bootstrap iterations on the training and test data sets with the estimated lower and upper CI. The table shows the comparison for each EEG devices and machine learning models.

For the Riemannian-based classifier, we observed a decrease in classification performance in the channel number configuration with 16 electrodes compared to 64 electrodes (at *p* < 0.05). We observed significantly reduced EEGNet classification performance for the dry-based compared to the gel-based EEG system.

When analyzing the two conventional multivariate linear classifiers that serve as a benchmark, we also found that higher classification performances could be achieved with gel-based EEG independent of channel number configuration [LDA-64-ch: 0.749; 95% CI (0.734, 0.764); SVM-64-ch: 0.773; 95% CI (0.759, 0.787); LDA-16-ch: 0.753; 95% CI (0.739, 0.768); SVM-16-ch: 0.757; 95% CI (0.743, 0.771)] compared with the dry-based EEG system [LDA: 0.665; 95% CI (0.643, 0.689); SVM: 0.676; 95% CI (0.654, 0.697)]. In addition, we observed a larger variance represented by larger CIs in the classification performances of all models for the dry-based compared with the gel-based EEG system.

### 3.4 Discussion study one

With the results of our first study, we showed the feasibility of decoding error related processes in response to the human observation of suboptimal robot action using data from different channel number configurations and EEG systems. To classify the ERPs related to error perception, we compared the performance of various machine learning models. These models included two linear benchmark models with conventional feature extraction (LDA and SVC), a Riemannian-based classifier, and a convolutional neural network (EEGNet).

In the context of previous work our models reached similar (Kim et al., 2017) or even higher classification performance (Iturrate et al., 2010, 2015; Ehrlich and Cheng, 2019)—especially for the EEGNet. Our results revealed that the classification performance of the convolutional neural network named EEGNet was superior to other models in all conditions (channel configurations and EEG systems). Despite observing a decline in decoding performance with the dry-based EEG system, the EEGNet was still able to achieve remarkably high classification performance surpassing chance levels and the linear benchmark models. Notably, EEGNet with dry-based EEG data outperformed averaged decoding performance reported in previous work (Iturrate et al., 2010, 2015; Ehrlich and Cheng, 2019). This is particularly promising because a high classification performance serves as a crucial prerequisite for a reinforcement learning system to acquire an optimal control policy (Sutton and Barto, 2018).

Hence, our results regarding the dry-based EEG system offer great potential for BCI applications and have practical implications, as the use of such systems significantly reduces setup effort compared to conventional gel-based systems, which typically require careful preparation of a larger number of electrodes. In addition to relatively high classification performances, we observed similar error-related potentials and SNRs for the dry-based compared with the gel-based EEG system (see Figures 3, 4). The observed N200 and delayed P300 over frontal and central electrodes were consistent with previous studies investigating erroneous and correct robot actions (Iturrate et al., 2015; Spüler and Niethammer, 2015; Kim et al., 2017; Ehrlich and Cheng, 2019). It demonstrates that both EEG systems are capable of capturing the characteristic ErrP waveform necessary for automatic classification within a BCI framework.

The high classification performance of EEGNet compared to other models might be attributed to its direct processing of pre-processed EEG time series, eliminating the need for explicit feature extraction (Lawhern et al., 2018). By utilizing depth-wise and separable convolutions, the model effectively captures both temporal and spatial information from the EEG signals. In a study conducted by Lawhern et al. (2018), the EEGNet outperformed conventional machine learning algorithms, such as a xDawn spatial filter combined with an elastic net regression, in within-subject classifications across various BCI paradigms. The authors advocate deep learning approaches like EEGNet due to their ability to strike a balance between input dimensionality and feature discovery. This characteristic is particularly advantageous as BCI technologies expand into new applications where suitable features remain uncovered (Schirrmeister et al., 2017; Lawhern et al., 2018). Deep learning models possess the capacity to effectively learn and extract valuable and robust features from high-dimensional EEG data. This ability, coupled with learning rate decay and the implementation of regularization techniques like dropout, proves advantageous in preventing the model from succumbing to overfitting induced by noisy patterns.

In conclusion, the findings from the first study demonstrate the effectiveness of both gel-based and dry-based EEG systems in capturing error related perception (ErrPs) and decoding suboptimal robotic behavior from the EEG signals. Particularly, the EEGNet model demonstrated superior performance, highlighting its potential as a reliable method for error perception analysis and decoding in both types of EEG systems. The comparative analysis is essential for establishing the validity of dry-EEG systems as a viable and efficient alternative, thereby advancing applicability and accessibility in future brain-computer interface applications.

## 4 Study two—feasibility study

### 4.1 Human feedback with deep reinforcement learning

In a second feasibility study, we investigated differences between an implicitly and explicitly trained version of the HF policy function and the effect on the performance of the deep RL + human feedback algorithm proposed by Akinola et al. (2020).

Thus, we implemented two versions of the proposed algorithm (for details see introduction):

1. Implicit version: Participants gave implicit feedback based on the automatic detection of perceived errors by the BCI.
2. Explicit version: Participants gave explicit feedback using a keyboard.

**Figure 6:**
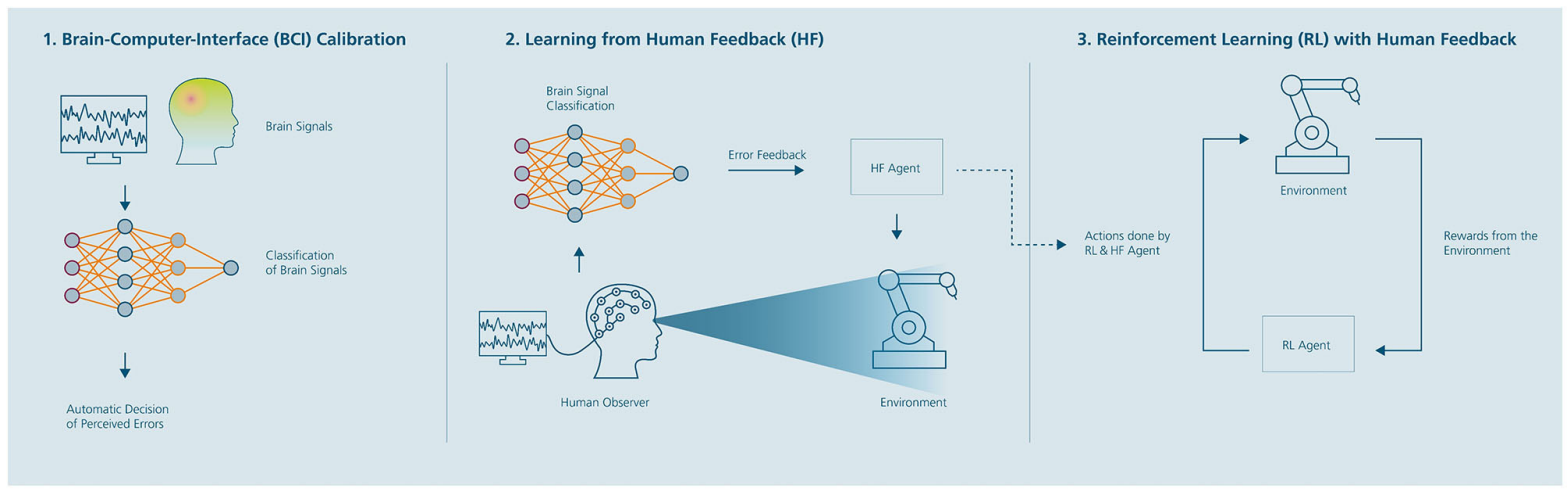
Deep reinforcement learning approach with learning from Brain-Computer Interface (BCI)-based human feedback. The approach consists of three stages: (1) Calibration of a BCI for automatic detection of erroneous (suboptimal) robot behavior by humans using electroencephalographic (EEG) signals; (2) Estimation of a reward function (human feedback policy) using the trained EEG-based BCI classifier; (3) learning a final RL strategy from sparse rewards, in which the human feedback (HF) policy guides the RL strategy. Adapted from Akinola et al. (2020).

The procedure for the explicit version follows the idea described in Christiano et al. (2017). Six participants were tested in the second study, with three training the HF policy function using implicit BCI-based feedback and three using explicit keyboard-based feedback. The deep RL + human feedback algorithm and procedure for the two versions of the implicit and explicit human feedback version can be summarized in three stages (see Figure 6).

Based on the findings from the first study, we employed the dry-EEG system for recording error related perception during the implicit BCI feedback. The procedure for data collection to calibrate the BCI (stage 1 from Figure 6) was equivalent to study one (see Section 3.1). The BCI predicted the perceived error perception of the participants in real-time. Overall, we recorded data pertaining to 400 single robot movements, with a fixed probability (50%) for erroneous actions from each participant. To train the BCI classifier we used EEGNet focusing on the time window of interest (200–800 ms) after the onset of end-effector movement. The real-time pre-processing of the EEG signals was the same as described in Section 3.2.1. The model was calibrated by splitting the dataset of each participant (400 trials) into training (70%), validation (15%), and test (15%) set. The validation set was intended for parameter optimization while the test set was used for final model performance evaluation using the ROC-AUC as metric. For training we used the same parameters as explained in Section 3.2.4.

In stage 2, the participant observed the robot agent performing random actions while trying to reach the goal. A full description of the procedure can be found in Supplementary Figures S4, S5. In the implicit BCI-based version the trained ErrP classifier was applied to the simultaneously recorded EEG signals to detect human feedback. Based on the implicit feedback, a supervised learning model was trained in real-time to predict the probability that an action will receive positive feedback (Akinola et al., 2020). Thus, the robot's strategy was continuously updated by maximizing the probability of success across all possible actions of the robot, i.e., HF policy. In addition to the implicit BCI, a HF policy was also trained with explicit input. For this, the HF policy was trained using the same procedure, but with feedback provided directly via keyboard input (button press, “*y*” for correct and “*n*” for incorrect behavior). In both cases, the training was done in real-time utilizing a fully connected neural network employing supervised learning, similar to Akinola et al. (2020). The network consisted of one hidden layer (32 units) with 8 input states and one output layer for 6 actions that is followed by a softmax-based classification block (see Supplementary Figure S6). The output of the hidden layer was fed into a rectified linear activation unit (ReLU). An epsilon-greedy strategy was chosen for training and selecting the robot's actions. The implementation was done in pytorch using the binary cross-entropy loss function in combination with the Adam algorithm as optimizer. Furthermore, the replay buffer adopted by Akinola et al. (2020) stored all past transitions, i.e., all agent experiences in a priority queue which were reused for training. Since each transition yielded information whether the transition results in a collision or not, we optimized the sampling of these transitions from the replay buffer such that each batch consisted of 10% collision and 90% non-collision samples (see Supplementary Figure S5). The idea behind this strategy was to reinforce the training behavior to avoid collisions. For comparison of the implicit and explicit version, a total of 1,000 feedback labels per participant were collected. To account for the possible problem of noisy BCI classification (Akinola et al., 2020), we also simulated noise in the explicit feedback with keyboard queries. Hence, participants trained two HF policy functions; a good explicit feedback version in which keyboard queries were received by the program as intended by the participant (100% accuracy) and a poorer explicit feedback version in which keyboard queries were received falsely with a 30% probability by the program (70% accuracy).

Finally, in stage 3, a robot agent is trained with the same task as in stage 2 but using a deep reinforcement learning algorithm where the agent is not rewarded directly by human feedback but by a reward learning condition (RL policy). To tackle the sparse reward problem, the previously trained HF policy models were used. Normally, the agent receives an observation from the environment and chooses an action based on the trained policy that maximizes the overall reward of an episode. Like in Deep Q-Learning (Sutton and Barto, 2018), an epsilon-greedy algorithm was deployed, but instead of a random action, the action suggested by the HF policy model was used. This feedback was used as the initial policy during the learning process toward the goal and thus increasing the chances of receiving positive rewards (Akinola et al., 2020). As learning progresses, the use of the HF policy was reduced while the use of the RL policy was increased as the behavioral strategy. The epsilon-greedy approach in which the HF policy was used starts with a probability of ε = 1 and decays linearly in the learning progress until it reaches ε = 0 at step count 125.000. After that, only the RL policy is used. A total of 8,000 episodes were trained per comparison with a maximum step count of 160 per episode. For evaluation, the success rate weighted by the normalized path length (SPL) was used (Anderson et al., 2018). We used the same architecture and hyperparameters of the deep reinforcement learning for both the implicit and explicit feedback version. In contrast to Akinola et al. (2020), we have chosen a deep deterministic policy gradient (DDPG) method as deep reinforcement learning. The implementation based on Lillicrap et al. (2015) is an adapted version from the open source repository,^5^ where the discount factor γ equals 0.9 and factor τ equals 0.005 for target network update. For the actor and critic network, the Adam optimizer was implemented with a learning rate of 0.003 and 0.001, respectively. To adapt DDPG for discrete action spaces, the output layer of the actor network was replaced with a softmax layer that produces a probability distribution over the possible discrete actions. Furthermore, an adapted replay buffer was used to store and reuse past transitions for training. In the replay buffer 10% of the batch contained transitions with the highest reward while the rest were randomly sampled. For each step, the model was updated 20 times. During an update each epoch contained a different randomly sampled training batch. The detailed architecture implemented in pytorch can be found in Supplementary Figure S7.

### 4.2 Results second proof-of-concept study

**Figure 7:**
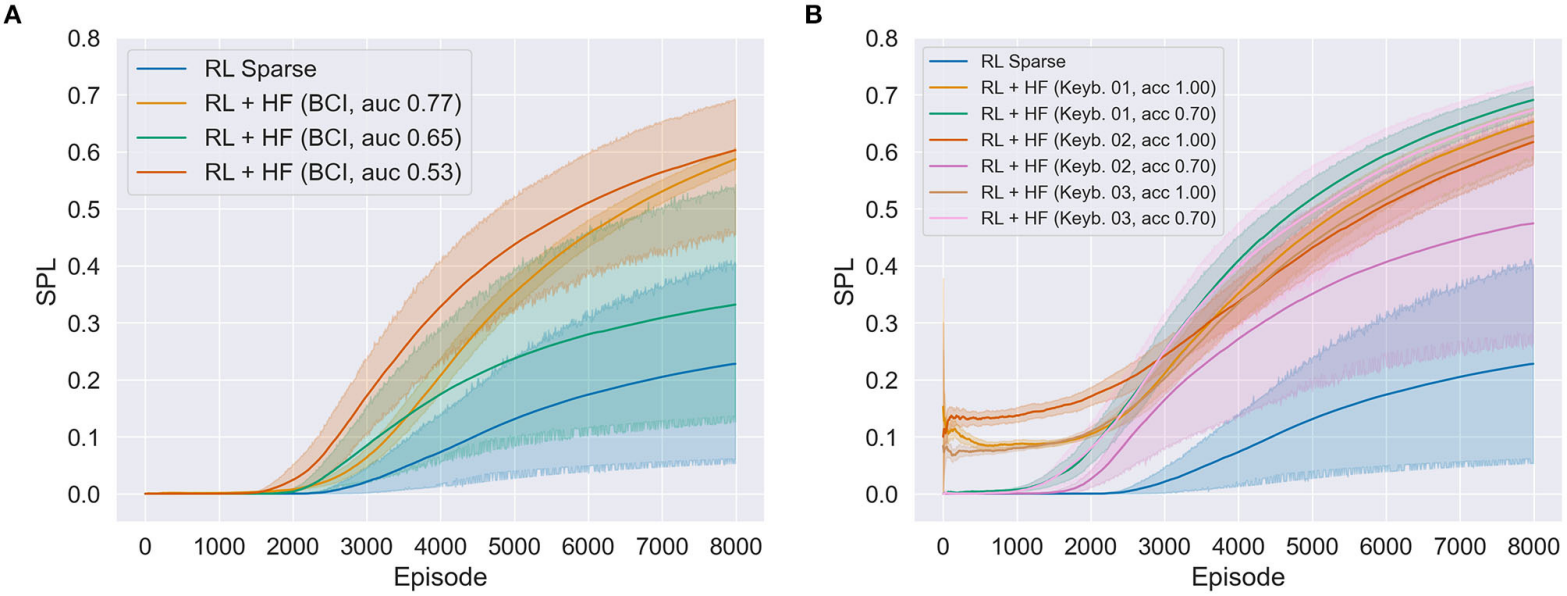
**(A)** Three successfully trained deep reinforcement learning models (using deep deterministic policy gradient method) with implicit Brain-Computer Interface (BCI)-based feedback from different participants with different BCI decoding performance as shown by the ROC-AUC metric and **(B)** six models with explicit feedback from three participants each with a good (Keyb. #, acc 100%) and poor (Keyb. #, acc 70%) version. The blue line shows the trained deep reinforcement learning model using only sparse rewards. In all the experiments, 10 models of 8,000 episodes were trained, where solid lines show the mean value estimated with bootstrapping and the shaded area the estimated 95% confidence interval (CI). The metric chosen was the success rate weighted by the normalized path length (SPL). RL, reinforcement learning; HF, human feedback.

In all the experiments, 10 reinforcement learning models of 8,000 episodes were trained. Mean values were estimated with bootstrapping (1,000 iterations) and corresponding 95% CIs were determined (see Figure 7). Three models were successfully trained based on implicit BCI-feedback from different participants [BCI, AUC 0.77: 0.587; 95% CI (0.570, 0.606); BCI, AUC 0.65: 0.332; 95% CI (0.137, 0.539); BCI, AUC 0.53: 0.603; 95% CI (0.459, 0.693)]. Six models were trained using explicit feedback from three participants. A model was trained with either a good (100%) or a poor (70%) variant of the feedback of one participant, thus resulting in six distinct models [Keyb. 01, ACC 1.00: 0.653; 95% CI (0.620, 0.679); Keyb. 1, ACC 0.70: 0.691; 95% CI (0.666, 0.715); Keyb. 02, ACC 1.00: 0.618; 95% CI (0.577, 0.655); Keyb. 02, ACC 0.70: 0.475; 95% CI (0.264, 0.658); Keyb. 03, ACC 1.00: 0.628; 95% CI (0.589, 0.658); Keyb. 03, ACC 0.70: 0.674; 95% CI (0.594, 0.724)]. In addition, one trained model was based only on sparse rewards from the environment [RL Sparse: 0.228; 95% CI (0.053, 0.405)]. In two versions of BCI-based HF policies the RL learning progress is remarkably accelerated (red and orange curves in Figure 7A). Compared to the model learning only through sparse rewards, better asymptotic learning performance was achieved by the explicit as well as the implicitly trained models. Both explicitly and implicitly trained models had exhibited accelerated learning relative to the sparse model. Moreover, two versions of BCI-based HF policies (red and orange curves in Figure 7A) showed similar asymptotic behavior to that achieved by explicitly trained model in which simulated noise was added through keyboard queries (70% accuracy; see Figure 5B). Comparing achieved accuracies of the implicit BCI-based models with the explicit models, it can be assumed that an implicit model with better accuracy would result in RL similar to the explicit model without noise which worked best in enhancing learning of the robot strategy. Overall, results illustrate that the variance of the RL process is reduced with increasing BCI accuracy. However, one of the BCI-based HF models was not good enough to train a useful HF policy and therefore accelerate the learning of the robot compared with the sparse model.

As expected, all three good versions (100%) of explicitly trained models showed the lowest variance and reached the target early in the learning process (although at the cost of many steps taken). It is important to note, that we observed no significantly better asymptotic learning behavior toward the end of the learning process (8,000th episode) when compared with the implicit version (see Figure 7A) and the version containing noise (see Figure 7B).

### 4.3 Discussion study two

The challenge of the robots learning the task is to avoid the obstacle wall and collisions with the robot arm to reach the goal as quickly and efficiently as possible. With our feasibility study in a realistic robot simulation environment we were able to extend the findings given in Akinola et al. (2020) with a systematic empirical comparison of an implicit vs. explicit human feedback policy version. Our results show that human feedback can be used to guide the robot agent toward optimal behavior more quickly than relying solely on trial-and-error exploration using sparse rewards. This is true for both versions of the proposed algorithm: Explicit (Figure 7B) and implicit (Figure 7A) given human feedback. Interestingly, the explicit version using 100% accurate feedback displays a learning effect earlier than the implicit version, thereby reaching the goal quickly at the cost of a longer path as shown by rather small SPL values. Moreover, comparing the implicit version with the noisy explicit version, we found no significant difference in the maximum learning rate as shown by the plateau of all model instances. Thus, the implicit HF policy works equally well in improving the learning rate of the reinforcement learning model as would a noisy explicit HF policy. The present results validate BCIs as implicit HF policies for reinforcement learning, showing a consistent improvement of the learning rate through human feedback, as well as the similarity of implicit feedback to explicit policies. Given that ideal explicit HF is not necessarily available, the implicit HF policy was proven to be a viable alternative to improve learning, a proposal that warrants further investigation in a larger cohort of participants. Due to the nature of a feasibility study having rather small sample sizes, we encourage other researchers to replicate our study to ensure the robustness of the observed findings. As a next step, we further plan to transfer this approach to more complex scenarios, e.g., in a dual-task scenario to answer further research questions: Can we implicitly detect and classify error-related brain potentials in a dual-task task and how is it dependent as a function of different mental load levels?

It is important to note, that our results replicate some of the results shown by Akinola et al. (2020) although we used another version of the deep RL algorithm. Deep deterministic policy gradient (DDPG; Lillicrap et al., 2015) is one of the earliest designed and most widely used algorithms that can operate on potentially large continuous state- and action spaces. It is an off-policy algorithm that is a variation of the Deep Q-Network (DQN; Mnih et al., 2015) algorithm which borrows the use of a replay buffer and target network learning both an actor function (also called policy) and a critic function. Some of the potential advantage of the DDPG over the PPO, as used in Akinola et al. (2020), is its performance for continuous action spaces. DDPG is specifically designed to handle continuous action spaces, while performing well in tasks that require precise and continuous control, such as robotic control tasks. PPO, on the other hand, is a more general algorithm that can handle both continuous and discrete action spaces but may not perform as well in environments with high-dimensional continuous action spaces (Lapan, 2018). It can also be assumed that DDPG might be more stable in future studies when training with larger and more realistic action spaces is needed. This is related to the fact that PPO uses a clipped surrogate objective function, which can lead to instability and slow convergence in high-dimensional action spaces, while in contrast DDPG uses a deterministic policy function and an off-policy actor-critic algorithm, which shows more robust performances in larger spaces (Lapan, 2018).

Another difference is the way on- and off-policy treat the usage of an replay buffer, which we further modified in our study as compared with Akinola et al. (2020). DDPG relies on experience replay to improve sample efficiency and reduce correlations in the training data (Lapan, 2018). This allows the algorithm to learn from past experiences and avoid overfitting to recent data. PPO, while it can also use experience replay, relies primarily on on-policy data collection, which can be less efficient and less effective in environments with sparse rewards. Overall, the advantages of off-policy methods include improved data efficiency, stable learning, and the ability to decouple exploration and exploitation. These characteristics of the off-policy DDPG would facilitate the transfer and usage of our findings in more realistic and continuous reinforcement learning action state space problems. We encourage further research in that direction to pave the way for more realistic applications.

Possible implications are the design of human-in-the-loop applications while interacting with robots (Salazar-Gomez et al., 2017; Xavier Fidêncio et al., 2022) or personalized AI systems to support and optimize machine decisions in (shared) autonomous vehicles or assistant interfaces for emergency situations (Shin et al., 2022; Wang et al., 2022). Another interesting application would be the usage in medical applications as training for a new generation of cognitive-assisted surgical robots (Wagner et al., 2021). The next generation of cognitive robots might learn during the interaction from implicitly generated human feedback via the BCI to give context-sensitive and individualized support, just as a human assistant would. Thus, through our approach, reward functions can be trained in a human-centered manner first in simulation and then transferred to real robots—sim-to-real transfer (Lapan, 2018).

Even though our results generally confirm that implicit HF policies work comparatively well to explicit feedback, for one participant, the implicitly trained model did not match the explicitly trained models. A possible reason for this could be that the participant was not as engaged in the task as the other participants, or possibly misunderstood the task and was actively employing a different cognitive strategy for providing feedback. This could have resulted in less pronounced ErrPs, thereby making a clear distinction between suboptimal (erroneous) and optimal (true) observed movements more difficult based on the EEG signals alone. Another possibility could be that the participant was generally not able to use the BCI modality of the study. There are several factors that influence the ability of a person to successfully use a BCI, for instance individual expertise or variability in brain structure (Becker et al., 2022). We encourage future work to include possible measures of variations in task performance of participants to systematically investigate potential reasons for performance differences of (implicitly) trained HF policies.

## 5 Conclusion

The first study showed that both gel-based and dry-based EEG systems were effective in detecting error-related perception and decoding robotic behavior from EEG signals. The EEGNet model was found to have high classification performance, suggesting that it could be dependably applied to error perception decoding in both gel- and dry-based EEG systems. We empirically showed that the EEGNet classifier in combination with the dry-based EEG-system provide a robust and fast method for automatically assessing sub- and optimal robot behavior. Through our second feasibility study we successfully demonstrated that the implicit BCI-based version significantly accelerates the learning process in a physically realistic and sparse simulation environment with even comparable performance to that achieved by explicit given feedback. Furthermore, the methodology is robust and rapidly applicable, as even suboptimal RF policies, like a BCI with low accuracy and a dry-based EEG system, can still manage to improve the learning.

## Data availability statement

The raw data supporting the conclusions of this article will be made available by the authors, without undue reservation.

## Ethics statement

The study protocol was approved by the Local Ethics Committee of the Medical Faculty of the University of Tuebingen, Germany (ID: 827/2020BO1). The studies were conducted in accordance with the local legislation and institutional requirements. The participants provided their written informed consent to participate in this study.

## Author contributions

MV: Conceptualization, Data curation, Formal analysis, Funding acquisition, Investigation, Methodology, Project administration, Writing – original draft, Writing – review & editing, Visualization, Software, Supervision, Resources, Validation. MB: Formal analysis, Methodology, Visualization, Writing – original draft, Writing – review & editing, Data curation, Investigation, Software, Validation. AV: Writing – original draft, Writing – review & editing, Visualization. KL: Formal analysis, Visualization, Writing – original draft, Writing – review & editing, Software.

## Footnotes

^1^ https://github.com/bulletphysics/bullet3

^2^ https://github.com/openai/gym

^3^ https://github.com/tensorflow/tensorflow/tree/v2.9.0/tensorflow/python

^4^ https://github.com/pytorch/pytorch

^5^ https://github.com/MrSyee/pg-is-all-you-need/blob/master/03.DDPG.ipynb
