# Supplementary material for: Combining brain-computer interfaces with deep reinforcement learning for robot training: a feasibility study in a simulation environment
Source: Front Neuroergon. 2023 Nov 23;4:1274730. doi: 10.3389/fnrgo.2023.1274730 (PMC10790930; doi:10.3389/fnrgo.2023.1274730)
Supplement: Supplementary file 1 [file Data_Sheet_1.PDF]

Supplementary Material

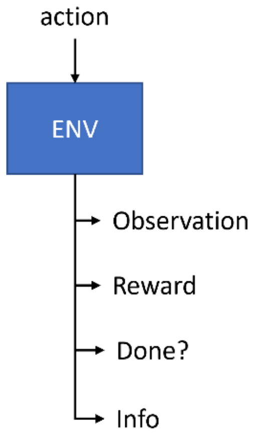

- Action
  - Defines step direction in which the robot moves the block
  - Is either left, right, forward, backward, up or down (6 directions)
  - Each direction axis has a different movement length in the world space

Direction	Left	Right	Forward	Backward	Down	Up
Length	-0.04	0.04	-0.02	0.02	-0.07	0.07

- Observation/state
  - Consist of world space coordinates and 5 laser observations
  - 3 cartesian coordinates (x, y, z) that are **normalized**
  - Output are float values between 0.0 and 1.0

State	X	Y	Z	L1	L2	L3	L4	L5
Val	[0, 1]	[0, 1]	[0, 1]	[0, 1]	[0, 1]	[0, 1]	[0, 1]	[0, 1]

Supplementary Figure 1: Detailed definition of the simulation environment for use in OpenAI Gym – definition of the continuous action space and observation (or state).

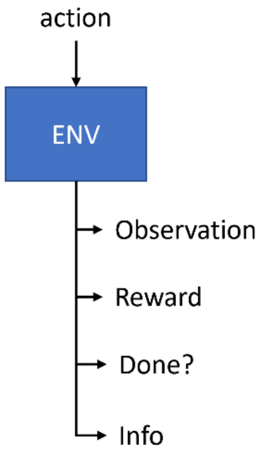

- Reward
  - A value corresponding to the reward of each step
  - Every step that doesn't guide to the goal has negative reward

- Done
  - Boolean: If True, an end-condition occured and the episode ends
  - End-Condition are Collisions/Out-of-bounds, reaching goal , and when maximum of 160 steps per episode is reached (over max steps)

End-Condition	Reward
Step	-0.05
Out of bounds / unreachable position	-8
Plane Collision	-8
Wall Collision	-8
Self Collision	-8
Over Max Steps	-7
Goal Reached	12

Supplementary Figure 2: Detailed definition of the simulation environment for use in OpenAI Gym – definition of reward and done condition (end of a learning episode).

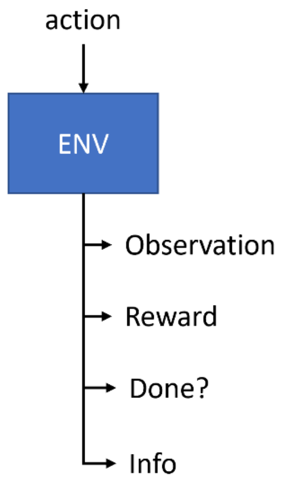

- Info
  - Contains additional information about the environment

Info	Description
Object Position	Cartesian Coordinate of the object
Path length	Calculated Shortest Path Length
Exit Reason	One of the End-Conditions
SPL	Success weighted by Path Length
Episodes	Current number of episodes

Supplementary Figure 3: Detailed definition of the simulation environment for use in OpenAI Gym – definition of the info.

Pipeline to train a Human Feedback (HF) Policy

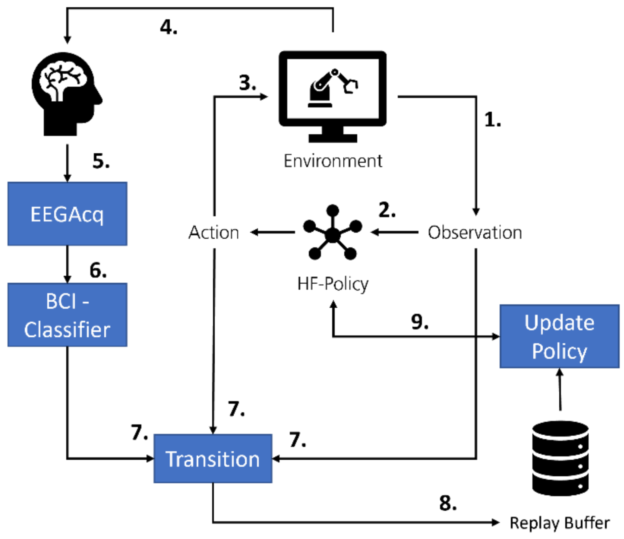

1. Environment gives a state or observation
2. HF Policy chooses an action depending on the observation given by the environment
3. Environment uses the action to move the robot
4. Stimuli created by the robot movement is observed by the participant
5. EEGAcq simultaneously records EEG signals and while doing real-time pre-processing and data epoching steps
6. BCI Classifier infers epoch data and predicts whether the movement seen by the participant was correct or incorrect
7. Action, Observation and Feedback is saved as a transition in the environment
8. Transition is pulled to a replay buffer
9. HF Policy is trained/updated using supervised learning and data from the replay buffer

Supplementary Figure 4: Detailed description of the real-time pipeline for supervised learning of a human feedback policy function (fully connected neural network).

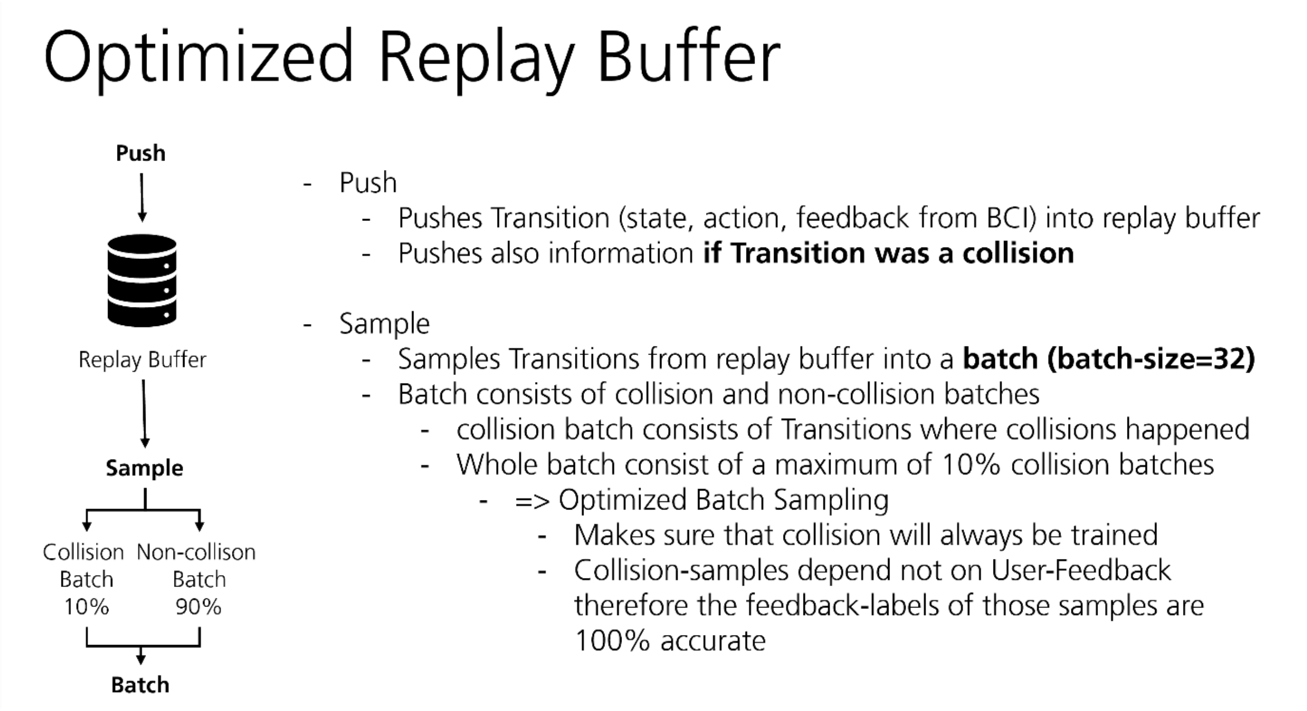

Supplementary Figure 5: Optimized replay buffer for training the human feedback policy via a fully connected neural network.

```
HFPolicyNet(
  (linear): Sequential(
    (0): Linear(in_features=8, out_features=32, bias=True)
    (1): ReLU()
    (2): Linear(in_features=32, out_features=6, bias=True)
    (3): Softmax(dim=1)
  )
)
```

Supplementary Figure 6: Fully connected neural network for the human feedback policy.

```
Actor(
  (net): Sequential(
    (0): BatchNorm1d(8, eps=1e-05, momentum=0.1, affine=True,
track_running_stats=True)
    (1): Linear(in_features=8, out_features=64, bias=True)
    (2): ReLU()
    (3): BatchNorm1d(64, eps=1e-05, momentum=0.1, affine=True,
track_running_stats=True)
    (4): Linear(in_features=64, out_features=64, bias=True)
    (5): ReLU()
    (6): BatchNorm1d(64, eps=1e-05, momentum=0.1, affine=True,
track_running_stats=True)
    (7): Linear(in_features=64, out_features=6, bias=True)
  )
)


Critic(
  (net): Sequential(
    (0): Linear(in_features=14, out_features=64, bias=True)
    (1): ReLU()
    (2): Linear(in_features=64, out_features=64, bias=True)
    (3): ReLU()
    (4): Linear(in_features=64, out_features=1, bias=True)
  )
)
```

Supplementary Figure 7: Deep deterministic policy gradient network architecture consisting of actor-critic neural networks.
